# Influence of a Biomass-Burning Event in PM_2.5_ Concentration and Air Quality: A Case Study in the Metropolitan Area of São Paulo

**DOI:** 10.3390/s21020425

**Published:** 2021-01-09

**Authors:** Gregori de Arruda Moreira, Izabel da Silva Andrade, Alexandre Cacheffo, Fábio Juliano da Silva Lopes, Alexandre Calzavara Yoshida, Antonio Arleques Gomes, Jonatan João da Silva, Eduardo Landulfo

**Affiliations:** 1 Federal Institute of São Paulo (IFSP), Campus Registro, São Paulo 11900-000, Brazil; 2 Center for Lasers and Applications (CELAP), Institute of Energy and Nuclear Research (IPEN), São Paulo 05508-000, Brazil; izabel.andrade@usp.br (I.S.A.); cacheffo@ufu.br (A.C.); fjslopes@unifesp.br (F.J.S.L.); yoshida@ufu.br (A.C.Y.); antonio.gomes@usp.br (A.A.G.); silva.jonatan@ufob.edu.br (J.J.S.); elandulf@ipen.br (E.L.); 3 Institute of Exact and Natural Sciences of Pontal (ICENP), Federal University of Uberlândia (UFU), Campus Pontal, Ituiutaba 38304-402, Brazil; 4 Department of Environment Sciences, Institute of Environmental, Chemical and Pharmaceutical Sciences (ICAQF), Federal University of São Paulo (UNIFESP), Campus Diadema, São Paulo 09913-030, Brazil; 5 Center for Exact Sciences and Technologies (CCET), Federal University of Western Bahia (UFOB), Campus Barreiras, Barreiras 47810-047, Brazil

**Keywords:** particulate matter, air quality, remote sensing, high order statistical moments, wildfires

## Abstract

Severe biomass burning (BB) events have become increasingly common in South America in the last few years, mainly due to the high number of wildfires observed recently. Such incidents can negatively influence the air quality index associated with PM_2.5_ (particulate matter, which is harmful to human health). A study performed in the Metropolitan Area of São Paulo (MASP) took place on selected days of July 2019, evaluated the influence of a BB event on air quality. Use of combined remote sensing, a surface monitoring system and data modeling and enabled detection of the BB plume arrival (light detection and ranging (lidar) ratio of (50 ± 34) sr at 532 nm, and (72 ± 45) sr at 355 nm) and how it affected the Ångström exponent (>1.3), atmospheric optical depth (>0.7), PM_2.5_ concentrations (>25 µg.m^−3^), and air quality classification. The utilization of high-order statistical moments, obtained from elastic lidar, provided a new way to observe the entrainment process, allowing understanding of how a decoupled aerosol layer influences the local urban area. This new novel approach enables a lidar system to obtain the same results as a more complex set of instruments and verify how BB events contribute from air masses aloft towards near ground ones.

## 1. Introduction

Air quality is one of the biggest problems faced by humanity currently, being more aggravated in large urban centers [1]. Among the various pollutants emitted daily in cities, particulate matter (PM) is noteworthy due to its relevant influence on the radiative balance [1,2,3,4,5], directly or indirectly, depending on its optical properties. The exposition to this class of pollutants is hazardous to human well-being [6], principally due to the exposition to PM_2.5_ (particles with aerodynamic diameter ≤ 2.5 µm). Because of their size, PM_2.5_ can enter in the respiratory tract and be deposited in the pulmonary region, causing respiratory and cardiovascular diseases [7,8]. Although vehicular traffic is an important source of PM_2.5_ in urban centers (contributing to consistent long-term degradation of urban air quality), external sources like biomass burning (BB) plumes can also contribute to the increase of the PM_2.5_ concentration, producing more serious adverse effects on urban air quality than vehicular traffic during short-term episodes of transported pollution from wildfires. Several studies have discussed the influence of BB episodes in the aerosol optical properties [9,10,11,12,13,14] and its relation with variations in the PM_2.5_ concentration and composition [2,9,15,16].

Wildfire activities are a common practice in general for those involved with agricultural and cattle breeding. Such practices have been extended and become common over the whole Amazon Region and its close neighboring areas, being considered the primary external anthropogenic sources of BB plumes in the Metropolitan Area of São Paulo (MASP), the most populous South America region. Air masses can advect such plumes into MASP, and they are more commonly detected between May and October [2]. MASP can be affected by BB events, especially during the dry season when dispersion conditions are worsened. In the last two decades, due to the employment of active remote-sensing systems, e.g., light detection and ranging (lidar), BB plumes can be detected in the atmosphere of MASP, in general up to 5000 m above ground level (a.g.l.) [11,14]. However, more studies addressing the influence of BB events on the air quality can be carried on, aiming to understand better and quantify the impact BB events caused by the pollutants [11], mainly when aerosol layers decoupled from the atmospheric boundary layer (ABL) are detected. In this situation, as the surface sensors cannot detect such layers, the effects of BB events on air quality can be underestimated. The high-spatial and temporal resolution capability of the lidar system recently have enabled the retrieval of important information of atmospheric evolution [17,18], for instance, the profile of aerosol distribution in the atmosphere every 2 s. In addition, measures with high acquisition rates allowed the implementation of high-order statistical moments analysis. The information provided by these statistical moments allows a better understanding of the vertical motion of the particle layers (skewness) and the variation of the mixing level in the ABL region (kurtosis) [17,18] so that the combination of this information can shed light on how the decoupled aerosol layers can interact with the ABL and can affect the concentration of pollutants near to the surface. Moreover, it is fundamental to identify the importance of some meteorological parameters, such as wind speed and direction, ABL height dynamics, and ventilation coefficient (VC), in order to understand better the dispersion process [19,20,21] and investigate the relationship with the variation in the PM_2.5_ concentration.

In this framework, this paper’s main objective is to evaluate the influence of a BB episode both on the air quality level and concentration of PM_2.5_ in the MASP. Such evaluation was performed by combining data from remote-sensing systems (multiwavelength Raman lidar, sunphotometer, and satellites), numeric model (hybrid single-particle lagrangian integrated trajectory model, HYSPLIT), radiosonde, and PM_2.5_ concentration data retrieved from surface instruments. In addition, to identify as a decoupled aerosol layer can influence the ABL region, the vertical movements and the mixing-level of aerosol plumes are described from high-order statistical moments generated by elastic lidar data. The BB event presented and analyzed here was detected in MASP on 23 July 2019.

The paper has the following structure: Section 2 gives a brief explanation of the experimental site and instruments used; Section 3 addresses the methodology; Section 4 gives the results and discussions; Section 5 draws conclusions.

## 2. Experimental Site and Instrumentation

### 2.1. Experimental Site

The measurement campaign was carried on in MASP, the most economically important region of Brazil (one of the ten biggest metropolitan regions in the world) with around 21 million inhabitants [22] and approximately 11 million vehicles [23], between 22 and 24 July 2019 (winter). The MASP has a humid subtropical climate, where the winter is dry and in general, with average temperatures slightly lower than those occurring during summer [24]. The thermal inversion layers, frequent in this season, inhibit the dispersion of pollutants. Such characteristics, combined with the lack of green areas, excess of asphalt, and buildings (considered heat island drivers), are responsible for intense air pollution episodes.

The large automobile fleet in MASP consists of light and heavy-duty vehicles and motorcycles, and it became the primary source of air pollutant emission [2]. According to the Environment Company of São Paulo State (CETESB), vehicle emissions on the MASP account for 97% of CO emissions, 76% of hydrocarbons, 64% of NO_X_, and 16,5% of SO_X_, 40% of PM_10_, and 37% PM_2.5_ concentrations [25].

### 2.2. Instrumentation

#### 2.2.1. São Paulo (SPU) Lidar Station System

The SPU Lidar Station system is a multiwavelength Raman lidar installed at the Nuclear and Energy Research Institute (IPEN/CNEN) in the city of São Paulo (23°33′ S, 46°38′ W, 760 m a.s.l., −3 UTC). This system is coaxial ground-based and operates with a pulsed Nd:YAG laser pointed towards the zenith direction with a repetition frequency of 10 Hz. The laser emits radiation at 355 nm, 532 nm, and 1064 nm. The SPU lidar system has three elastic channels (355 nm, 532 nm, and 1064 nm) and three Raman channels (387 nm, 408 nm, and 530 nm) [18]. The system reaches full overlap at 300 m a.g.l. and operates with a spatial and temporal resolution of 7.5 m and 2 s, respectively.

#### 2.2.2. Aerosol Robotic Network (AERONET)

The Aerosol Robotic Network (AERONET) is a ground-based monitoring network dedicated to monitoring and characterizing aerosols on a local and global scale [26]. The data of aerosol optical depth (AOD) and Ångström exponent (AE) used in this work were obtained from the data level 1.5 of the AERONET São Paulo Station (23°33′ S, 46°38′ W, 786 m a.s.l.). In order to present the AOD and AE data with a temporal resolution of one hour, the hourly average was calculated for each variable. The standard deviation will be presented together with the average values.

#### 2.2.3. QUALAR Platform and Monitoring Stations

CETESB is the company responsible for monitoring the air quality in São Paulo State. It has 63 monitoring stations distributed statewide, which provide information on meteorological parameters (surface temperature and humidity, wind speed, others), pollutant concentration (PM_10_, PM_2.5_, CO_2_, others), and air quality classifications and indexes. This information is available in the QUALAR system (https://qualar.cetesb.sp.gov.br/) [23].

In this paper, PM_2.5_ concentration and horizontal wind speed values were retrieved, with 1-h time resolution, at the Pinheiros Station (23°33′ S, 46°39′ W, 760 m a.s.l.). Considering the CETESB stations that render data about PM_2.5_, Pinheiros is the closest station to IPEN (3.5 km) with available data in the analyzed period. The air quality index (AQI) classifications applied by CETESB are based on CONAMA (Brazilian Environment Council) Resolution 491/2018 [25]. The AQI were obtained from all PM_2.5_ stations of MASP with available data in the analyzed period.

#### 2.2.4. Hybrid Single-Particle Lagrangian Integrated Trajectory Model (HYSPLIT)

The HYSPLIT [27] is a hybrid model between Lagrangian (which is applied to calculate the advection and diffusion as backward trajectories of the air parcels) and Eulerian (which is applied to compute the pollutant air concentrations using a fixed three-dimensional grid as a frame of reference) approaches. These combined methodologies allow such a model to compute air parcel trajectories, complex transport, dispersion, deposition, and chemical transformation. In this paper, HYSPLIT was applied to estimate the backward trajectories of air parcels, with a temporal resolution of 3 h, that arrived in the MASP on 23 July 2019.

#### 2.2.5. Radiosonde

At the Campo de Marte Airport (23°30′ S, 46°38′ W, 760 m a.s.l.), the radiosondes were launched twice daily (00:00 UTC and 12:00 UTC). The data are available at the University of Wyoming website (http://weather.uwyo.edu/upperair/sounding.html).

#### 2.2.6. Wildfires Database

The Wildfires Database (https://queimadas.dgi.inpe.br) is coordinated and operated by the Brazilian National Institute for Space Research (INPE). Information and data on burning episodes in South America are available in this database. Such information is obtained from data from different satellites. In this work, we employ data from the satellites Geostationary Operational Environmental Satellite 16 (GOES-16), through its Advanced Baseline Imager (ABI), and Aqua, through its Moderate Resolution Imaging Spectroradiometer (MODIS) [28].

## 3. Methodology

### 3.1. Atmospheric Boundary Layer Height Retrieval

The ABL can be subdivided into some main sublayers: convective boundary layer (CBL), stable boundary layer (SBL), and residual layer (RL), which are directly associated with the stability variation during the daily cycle of this layer [29]. Due to the radiative cooling of the ground during the night, the SBL is formed. This layer is warmer than the underlying surface and suppresses the turbulence mixing. The SBL is topped by the RL, which has a uniform potential temperature profile with a value higher than the layer below. This layer also displays a weak and sporadic turbulence and has both the pollutants remaining and the maximum height of the CBL of the previous day. These two often decoupled layers coexist until the moment where, due to sunrise, the heat transfer, caused by the warm ground, generates convective thermals that cause turbulence mixing, substituting the SBL with the CBL. Into the CBL, the vertical distribution of potential temperature is uniform. The CBL grows during the day (breaking the RL), reaching the maximum height around the middle of the day.

Due to the limitation in the measurement time, the CBL height (CBLH) and RL height (RLH) were estimated from radiosonde and lidar data.

#### 3.1.1. Residual Layer Height (RLH) and Convective Boundary Layer Height (CBLH) Determined from Light Detection and Ranging (Lidar) Data

Visible (532 nm) lidar observations are used for the analysis. Before implementing the algorithms described below, three corrections are performed to guarantee the quality of the data. Firstly, the dark current signal (DC(z)) is subtracted from the raw signal (P(z)) in order to minimize the influence of the electronic noise. Next, the background radiation signal (BG) is removed to reduce the influence of external sources, e.g., the Sun. Following this, each profile point generated using the previous steps is multiplied by its squared correspondent height (due to the attenuation of the signal with the height), resulting in the range corrected signal (${\mathrm{RCS}}_{532}$):

(1) $${\mathrm{RCS}}_{532}\left(z\right)=\left(P\left(z\right)-\;\mathrm{DC}\left(z\right)-\mathrm{BG}\right)*z^{2}$$

The CBLH was estimated with the wavelet covariance transform method (WCT) because this method enables the CB to identify the CB even when the RL is present. [30]. The WCT calculates the covariance between ${\mathrm{RCS}}_{532}$ and a mother-wavelet, given by the Haar function, denoted by $h\left(\frac{z-b}{a}\right)$. The covariance profile maximum corresponds to the keenest reduction in the ${\mathrm{RCS}}_{532}$. This reduction is associated with a high decrease in the aerosol concentration and, consequently, the top of the CBL. Hence, such height is selected as the CBLH, according to the following equations:

(2) $$\mathrm{CBLH}=\mathrm{Max}\left(W\left(a,b\right)\right),\;W\left(a,b\right)=\;\frac{1}{a}\int_{z_{i}}^{z_{f}}\bar{{\mathrm{RCS}}_{532}}\left(z\right)h\left(\frac{z-b}{a}\right)\mathrm{dz},$$

where a and b are the dilatation and transition coefficients linked to the mother-wavelet, z is the height above the ground and, $z_{i}$ and $z_{f}$ are the lower and upper limits of the $\bar{{\mathrm{RCS}}_{532}}\left(z\right)$.

The variance method (VM) was adopted to determine the RLH [31]. This method computes the top of the aerosol layer based on the maximum variance in the ${\mathrm{RCS}}_{532}$ observed during a determined time-interval, which in this paper is 10 min, in order to reduce the influence of the noise at the signal. The VM takes into account the equations:

(3) $$\mathrm{RLH}=\mathrm{Max}\left(\sigma_{{\mathrm{RCS}}_{532}}^{2}\left(z\right)\right),\;\sigma_{{\mathrm{RCS}}_{532}}^{2}\left(z\right)=\frac{1}{n}\;\overset{n}{\underset{i=1}{\sum }}{\left[{\mathrm{RCS}}_{532_{i}}\left(z\right)-\bar{{\mathrm{RCS}}_{532}}\left(z\right)\right]}^{2},\;\;\;$$

where z is the height and n and $\bar{{\mathrm{RCS}}_{532}}\left(z\right)\;$are the number and the average value of ${\mathrm{RCS}}_{532}$ profiles, respectively, observed during a 10-min time interval. Because of the entrainment of clean air coming from the free troposphere (FT) in the ABL during the convective period, the maximum of $\sigma_{{\mathrm{RCS}}_{532}}^{2}\left(z\right)$ occurs at the top of the CBL, in a clear sky situation. During the early morning, the RL can generate secondary sharp peaks in the RCS, causing a misinterpretation in the CBLH estimated so that often the RLH is detected. Therefore, the variance method estimates the RLH until the difference between this height and the height estimated by the WCT method is lower than 100 m.

#### 3.1.2. RLH and CBLH Estimated from Radiosonde Data

From the radiosonde at 12:00 UTC, the RLH and CBLH are estimated based on the potential temperature and relative humidity (only for CBLH) profiles until 3500 m. At first, CBLH is evaluated using the parcel method [32]. Such a method estimates the height where an air portion in ambient temperature can rise adiabatically from the ground by the convective process. The CBLH estimation is reinforced by detecting the height where the minimum vertical gradient in the relative humidity profile occurs [33]. The RLH is identified from the high gradient in the potential temperature profile above the CBLH. In the radiosonde data at 00:00 UTC, only the RLH is estimated based on the same technique applied at 12:00 UTC.

### 3.2. Ventilation Coefficient

An essential parameter to evaluate the air pollutants’ dispersion level is the VC, expressing the rate at which the air in the CBL is transported away from a determined region [31]. The VC is calculated from the combination of the CBLH and horizontal wind speed (WS). In this paper, the VC is estimated from the 1-h average values of the horizontal wind speed ($\bar{\mathrm{WS}}$), and CBLH ($\bar{\mathrm{CBLH}}$) calculated from lidar data, obeying the equation:

(4) $$\mathrm{VC}=\;\bar{\mathrm{CBLH}}*\bar{\mathrm{WS}}.$$

Consequently, such a variable is obtained with a temporal resolution of one hour.

### 3.3. High-Order Statistical Moments

The high frequency of lidar data acquisition (2 s) allows the high-order statistical moments to be estimated until the fourth-order [17,18]. Such moments are obtained from the fluctuation of ${\mathrm{RCS}}_{532}$ (${{\mathrm{RCS}}^{\prime }}_{532}$), which is obtained from the Reynolds decomposition:

(5) $${{\mathrm{RCS}}^{\prime }}_{532}{\left(z\right)}_{i}=\;\bar{{\mathrm{RCS}}_{532}}\left(z\right)-{\mathrm{RCS}}_{532}{\left(z\right)}_{i},$$

where $\bar{{\mathrm{RCS}}_{532}}\left(z\right)$ denotes the average value obtained for one hour of measurements.

In this paper, the high order statistical moments explored are skewness and kurtosis of the ${\mathrm{RCS}}_{532}$. The skewness can provide information regarding the aerosol plumes’ vertical movements since the positive values are associated with updrafts and negative ones with downdrafts. Kurtosis is linked to the level of mixing. Values lower than 3.0 for kurtosis are related to a well-mixed CBL, and values higher than 3.0 are related to a low-mixed CBL [17,18].

## 4. Results and Discussion

### 4.1. First Day (One Day before the Biomass Burning (BB) Event)

**Figure 1:**
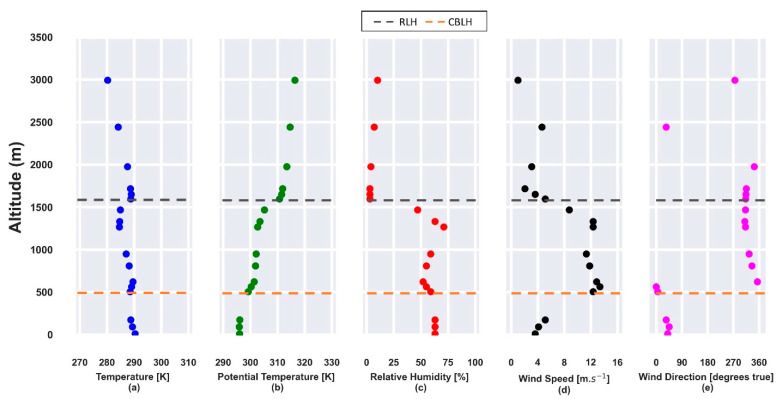
Atmospheric parameters obtained from radiosonde data on 22 July 2019 at 12:00 UTC. (**a**) Temperature [K]; (**b**) potential temperature [K]; (**c**) relative humidity [%]; (**d**) horizontal wind speed [m·s^−1^]; (**e**) wind direction [degrees true]. The gray and orange dashed lines represent the residual layer height (RLH) and convective boundary layer height (CBLH), respectively.

Figure 1 presents the profiles obtained from radiosonde launched on 22 July 2019. The vertical profiles of potential temperature and relative humidity, obtained at 12:00 UTC, show the CBLH and RLH at approximately 500 m and 1550 m, respectively (Figure 1b,c) so that both layers are trapped by a thermal inversion (Figure 1a). In the CBL region, the relative humidity (RH) is lower than 70%. The horizontal wind speed profile (Figure 1c) has values higher than 10 m·s^−1^ and the predominance of a NNW direction between 500 m and 2000 m.

**Figure 2:**
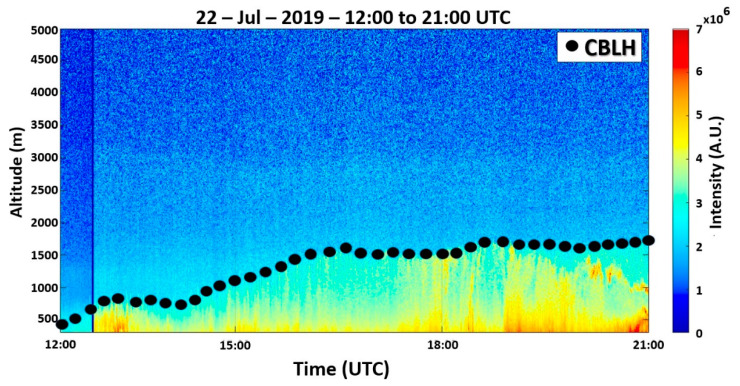
Range corrected signal (${\mathrm{RCS}}_{532}$) profiles from 12:00 UTC to 21:00 UTC on 22 July 2019. The black dots represent the CBLH. The color bar represents the intensity of the ${\mathrm{RCS}}_{532}$.

Figure 2 presents the ${\mathrm{RCS}}_{532}$ profiles, obtained from 12:00 UTC to 21:00 UTC, where an ABL without the presence of decoupled sublayers can be observed. At the start of the measurement, the CBLH (550 m) is higher than the value obtained from radiosonde data (500 m). The RL cannot be detected. A low growth regime is observed until approximately 14:00 UTC when the CBL growth rate increases and the CBLH reaches its maximum value (around 1700 m) at 19:00 UTC. Then, it does not have significant variation (around 1750 m) until the end of the measurement.

**Figure 3:**
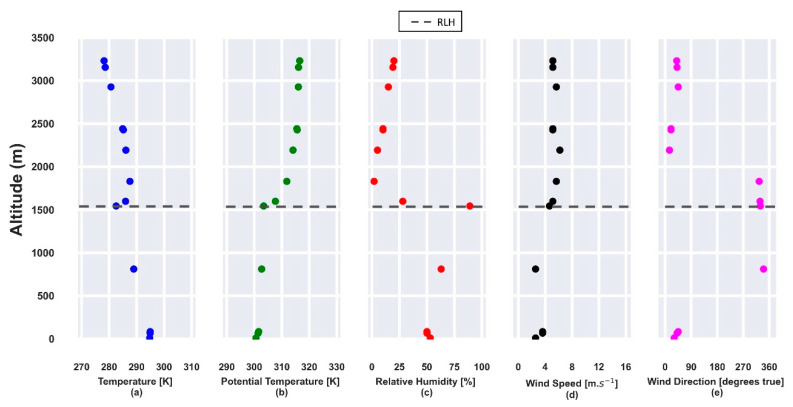
Atmospheric parameters obtained from radiosonde data on 23 July 2019 at 00:00 UTC. (**a**) Temperature [K]; (**b**) potential temperature [K]; (**c**) relative humidity [%]; (**d**) horizontal wind speed [m·s^−1^]; (**e**) wind direction [degrees true]. The gray dashed line represents the RLH.

The RL height, obtained from radiosonde data, at 00:00 UTC (23 July), Figure 3b, is situated around 1530 m and trapped by a thermal inversion (Figure 3a). The RH is around 50% in the first few meters (Figure 3c), increasing to values higher than 75% above the RLH. Between 2000 m and 3000 m, the horizontal wind speed has values higher than 5 m·s^−1^ (Figure 3d) with NWE and NE directions (Figure 3e).

**Figure 4:**
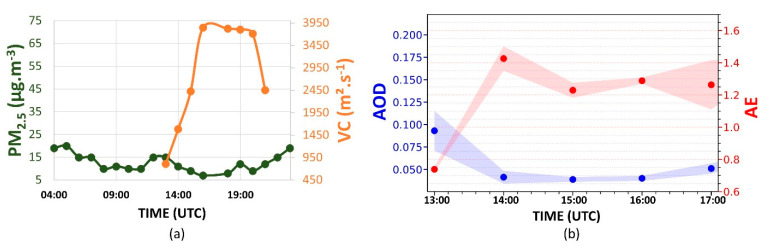
(**a**) Daily cycles of PM_2.5_ concentration (green dots) and ventilation coefficient (VC, orange dots); (**b**) aerosol optical depth (AOD) at 500 nm (blue points) and Ångström exponent (AE, 440/675) (red points) hourly averages variation from 13:00 UTC to 17:00 UTC, on 22 July 2019. The blue and red shadows represent the AOD and AE standard deviation, respectively. The Aerosol Robotic Network (AERONET) data level is 1.5 for AOD and AE.

Figure 4a displays the daily variation of PM_2.5_ concentration. Between 05:00 to 08:00 UTC, the PM_2.5_ concentration decreases from 21 to 9 µg·m^−3^. At 09:00 UTC, an increase (10 µg·m^−3^) can be observed. However, at 10:00 UTC, the concentration decreases again, reaching 7 µg·m^−3^ at 11:00 UTC. After this period, the concentration begins to rise. This increase occurs due to vehicle emissions during the rush hour in the morning, as indicated by [34]. However, at 13:00 UTC, the PM_2.5_ concentration begins to decrease again, reaching 6 µg·m^−3^ at 16:00 UTC. It is a consequence of CBLH growth, which results in a VC increasing (3900 m^2^·s^−1^), favoring the dispersion process. At 18:00 UTC, the VC begins to decrease, significantly reducing between 20:00 UTC and 21:00 UTC (2450 m^2^·s^−1^). From 20:00 UTC, the PM_2.5_ concentration increases continuously, reaching 20 µg·m^−3^ at 23:00 UTC. The transition from unstable to stable behavior in ABL (where there are practically no convective movements, with a predominance of mechanical turbulence) and the thermal inversion situated above the RL contribute together to reducing the efficiency of the dispersion of the pollutants.

Figure 4b presents the hourly-variation of AE (red dots) and the AOD (blue dots) between 13:00 UTC and 17:00 UTC. The increase in the AE between 13:00 and 14:00 UTC (0.73 to 1.42) indicates a reduction in the aerosols’ average size in the atmospheric column. Such a variation is accompanied by a reduction in the average AOD values (0.900 to 0.045). After 14:00 UTC, the AE decreases to lower values (indicating an increase in the aerosols’ average size in the atmospheric column), reaching 1.27 at 17:00 UTC. The AOD has a low variation after 14:00 UTC, reaching 0.050 at 17:00 UTC.

On this day, the AQI of PM_2.5_ was classified as good (≤25 µg·m^−3^) at *Pinheiros* station, with an average daily concentration of 16 µg·m^−3^.

### 4.2. Second Day (Detection of the BB Event)

**Figure 5:**
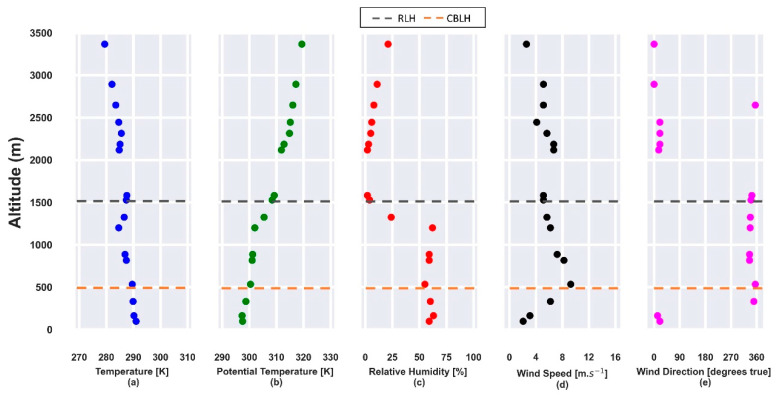
Atmospheric parameters obtained from radiosonde data on 23 July 2019 at 12:00 UTC. (**a**) Temperature [K]; (**b**) potential temperature [K]; (**c**) relative humidity [%]; (**d**) horizontal wind speed [m·s^−1^]; (**e**) wind direction [degrees true]. The gray and orange dashed lines represent the RLH and CBLH, respectively.

The potential temperature and RH profiles, obtained from radiosonde data at 12:00 UTC, reveal the CBLH and RLH at approximately 500 m and 1500 m (Figure 5b,c), respectively, and the last one is trapped by a thermal inversion (Figure 5a). In the region below the RLH, the RH (Figure 5c) presents slight variability with an average value of around 60%. Two maximum peaks can be observed in the horizontal wind speed profile (Figure 5d) at 500 m and 2200 m, with a predominance of NNW and N directions, respectively (Figure 5e).

**Figure 6:**
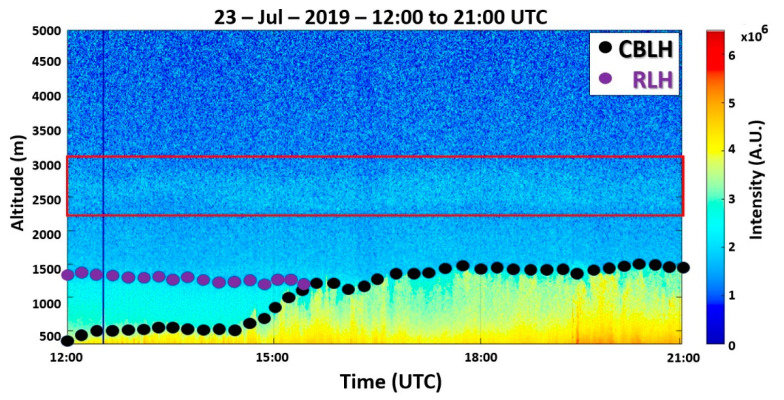
${\mathrm{RCS}}_{532}$ profiles from 12:00 to 21:00 UTC on 23 July 2019. The black dots represent the CBLH, and the purple dots represent the RLH. The color bar represents the intensity of the ${\mathrm{RCS}}_{532}$.

The ${\mathrm{RCS}}_{532}$ profiles (Figure 6), obtained between 12:00 UTC to 21:00 UTC, present the ABLH behavior, where can be observed an RLH varying from 1450 m to 1300 m, from the beginning of measurement until approximately 15:30 UTC. The CBLH has low variations in the first hours of measurement, with a rapid increase between 14:40 to 15:40 UTC, reaching 1470 m. Around 18:00 UTC, the CBLH reaches its maximum height (1500 m). After that, it remains practically constant.

**Figure 7:**
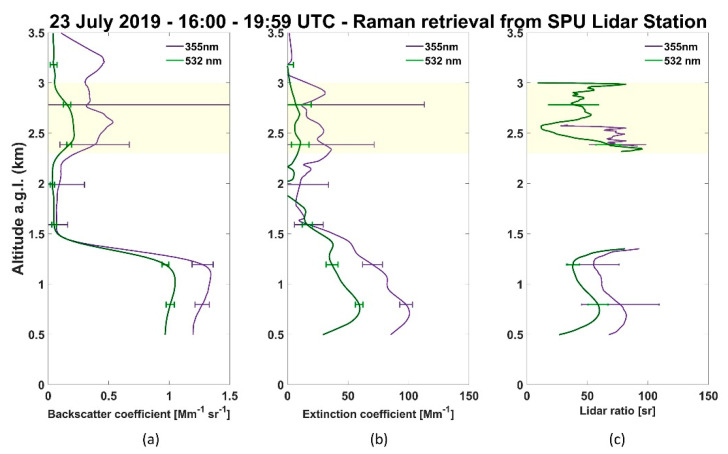
Profiles of the backscatter (**a**) and extinction (**b**) coefficients, and light detection and ranging (lidar) ratios (**c**) retrieved on 23 July 2019, from 532 nm and 355 nm channels, between 16:00 UTC and 20:00 UTC.

The profiles of the aerosols’ optical properties were retrieved for this day by applying Raman inversion analysis [35,36]. Figure 7a,b show the backscatter and extinction profiles, respectively, from the wavelengths 532 nm (green line) and 355 nm (violet line), where an aerosol layer between 2500 m and 3000 m can be noticed clearly due to an increase in the values of the backscatter and the extinction coefficient profiles. The lidar ratio profiles (Figure 7c) show two distinct layers: the first one is related to the CBL, and the second one is the decoupled aerosol layer between 2500 m to 3000 m. For the CBL, there were observed mean lidar ratio values of (48 ± 9) sr and (69 ± 10) sr, at wavelengths 532 nm and 355 nm. Lidar ratio values of (50 ± 34) sr at 532 nm and (72 ± 45) sr at 355 nm were retrieved for the decoupled layer. According to the literature, such lidar ratio values agree with aerosols profiles originated from biomass burning events [37,38,39]. Nicolae et al. [40] reported biomass burning fresh aerosol classification with lidar ratio values of (45.7 ± 6.4) sr and (73.0 ± 11.6) sr for 532 nm and 355 nm, respectively. In our case, since the aerosol optical properties were retrieved by applying the Raman technique during daytime measurements, which implies profiles with considerable background noise, the lidar ratio values for the decoupled aerosol layer presented large uncertainties.

**Figure 8:**
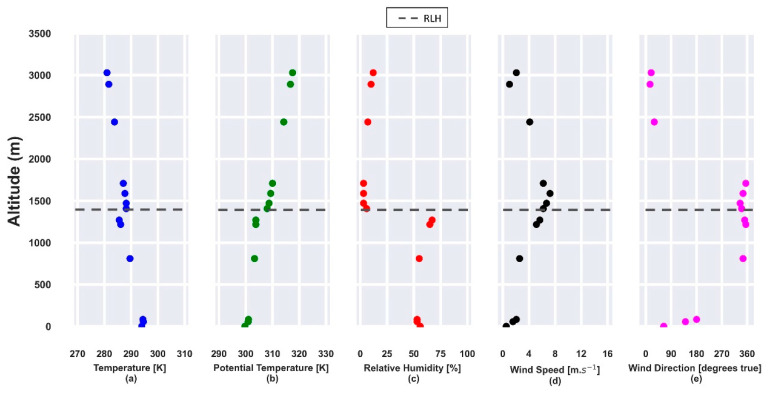
Atmospheric parameters obtained from radiosonde data on 24 July 2019 at 00:00 UTC. (**a**) Temperature [K]; (**b**) potential temperature [K]; (**c**) relative humidity [%]; (**d**) horizontal wind speed [m·s^−1^]; (**e**) wind direction [degrees true]. The gray dashed line represents the RLH.

From the potential temperature and RH profiles (Figure 8 b,c), obtained from radiosonde data at 00:00 UTC (24 July), it is possible to observe the top of the RL, at about 1470 m, which is confined by a thermal inversion (Figure 8a). From the surface until 800 m, the RH (Figure 8c) is around 53%, reaching higher values (around 70%) at the top of RLH. The horizontal wind speed profile has a maximum between 1500 m and 2000 m (Figure 8d), with a predominant NNW direction (Figure 8e).

**Figure 9:**
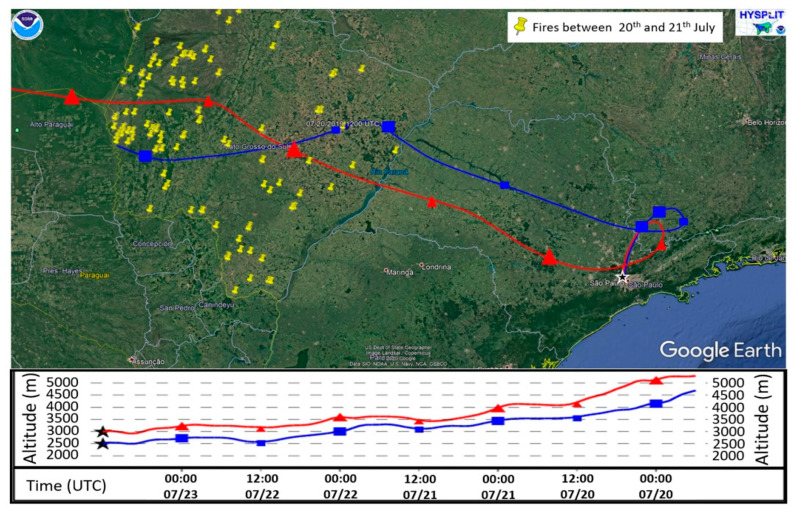
The 96-h backward trajectories generated from the hybrid single-particle lagrangian integrated trajectory model (HYSPLIT). The yellow points represent the wildfires detected by the satellites GOES-16 and Aqua in Brazil’s Midwest region between 20 and 21 July. The blue and red lines indicate the backward trajectories of the air parcels that arrived in the Metropolitan Area of São Paulo (MASP) at 2500 m and 3000 m, respectively. The map was generated using Google Earth.

The yellow points in Figure 9 represent wildfire episodes detected by the satellites GOES-16 and Aqua between 20 and 21 July 2019, in the Brazilian center-west region. The backward trajectories demonstrate the route traveled by the aerosol plumes that arrived at 2500 m (blue line) and 3000 m (red line) in MASP on 23 July 2019, at 12:00 UTC. The trajectories are coincident with wind direction (NNW) 2500 and 3000 m presented in Figure 5e. The analysis of the backward trajectories, the height of the decoupled aerosol layer (Figure 6), and the lidar ratio values (Figure 7c) reinforce the assertion that that aerosols originated from wildfires compose the decoupled layer.

**Figure 10:**
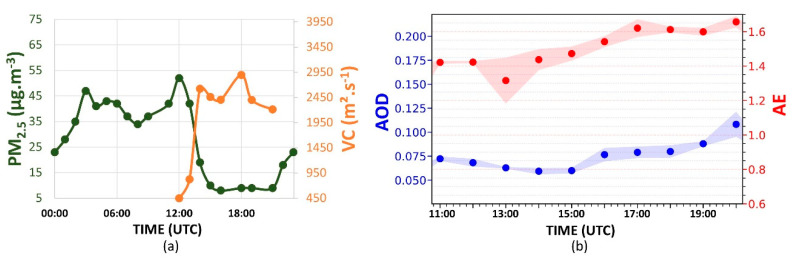
(**a**) Daily cycles of PM_2.5_ concentration (green dots) and VC (orange dots); (**b**) AOD at 500 nm (blue points) and AE (440/675) (red points) hourly averages variation from 11:00 UTC to 20:00 UTC, on 23 July 2019. The blue and red shadows represent the AOD and AE standard deviation, respectively. The AERONET data level is 1.5 for AOD and AE.

The PM_2.5_ concentration (Figure 10a) increases between 00:00 UTC and 04:00 UTC (25 to 46 µg·m^−3^), presenting some variations from 05:00 UTC and increasing significantly between 11:00 UTC and 12:00 UTC, due to rush hours (as observed in the early day), reaching 54 µg·m^−3^. At 12:00 UTC, when the CBL begins to rise, the values of VC (Figure 10a) increase significantly (reaching 2450 m^2^·s^−1^ at 14:00 UTC), and the PM_2.5_ concentration begins to decrease, reaching the minimum (7 µg·m^−3^) value at 16:00 UTC. Although the CBL continues rising until around 18:00 UTC, the low wind speed values inhibit the VC increase, occasioning low values (2900 m^2^·s^−1^) compared to the early day (3900 m^2^·s^−1^). At 18:00 UTC, when the VC values begin to decrease continuously, the PM_2.5_ concentration increases slightly, with a significant variation (6 to 17 µg·m^−3^) between 20:00 UTC and 21:00 UTC (beginning of the stable period in the atmosphere).

The AE value (Figure 10b) always remains above 1.35, so that after 13:00 UTC, it continually increases, reaching 1.65 at 20:00 UTC. Such a pattern demonstrates the predominance of particles with a smaller size than the particles observed in the previous day, where the average value of AE was 1.20. In addition, the AE values observed on 23 July follow those observed in the BB events (>1.3) in other regions and from the literature [37,38,39]. Compared to the previous day (where the average value was around 0.05), the higher AOD value indicates an increase in the aerosol load, mainly after 14:00 UTC, when the AOD is continuously increasing, reaching 0.11 at 20:00 UTC. Such variations are not detected directly by the CETESB stations because their detections are limited at the surface level. Nevertheless, the sun photometers obtain data from the entire atmospheric column [26] so that AOD and AE data are more sensitive to the effects between 2500 m and 3000 m.

**Figure 11:**
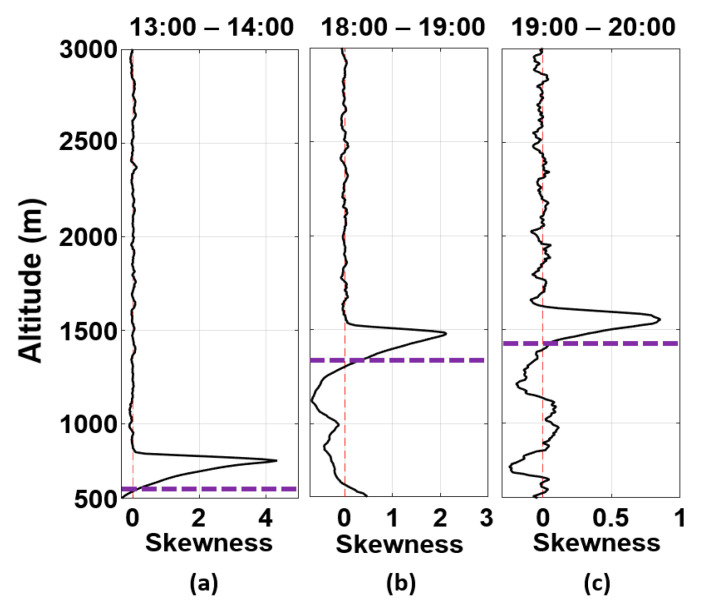
${\mathrm{RCS}}_{532}$ skewness profiles obtained on 23 July 2019, between (**a**) 13:00–14:00 UTC, (**b**) 18:00–19:00 UTC, (**c**) 19:00–20:00 UTC. The purple dotted line represents the CBLH.

**Figure 12:**
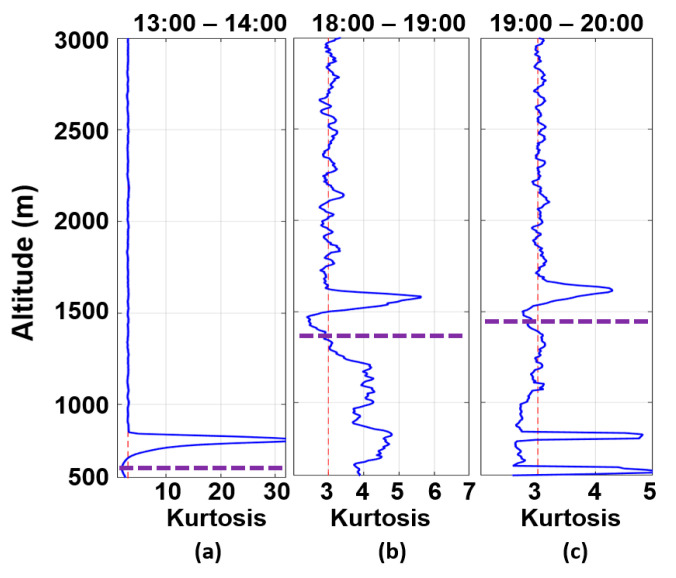
${\mathrm{RCS}}_{532}$ kurtosis profiles obtained on 23 July 2019, between (**a**) 13:00–14:00 UTC, (**b**) 18:00–19:00 UTC, (**c**) 19:00–20:00 UTC. The purple dotted line represents the CBLH.

However, considering the ABL behavior, it is possible that, due to the entrainment processes, the aerosols of the decoupled plume gradually enter the ABL, thus being able to reach its lowest region after a determined time interval. Such a phenomenon can be observed by the combination of the skewness (Figure 11) and kurtosis (Figure 12) profiles obtained from ${\mathrm{RCS}}_{532}$, which provide information about the vertical movement of aerosol plumes and the mixing level of the ABL, respectively [17,18].

As expected, between 13:00 UTC and 14:00 UTC (Figure 11a), the CBLH is situated in the inflection point of the skewness profile, which has a predominance of negative values (downdrafts) in the CBL region and positive values (updrafts) above it [17,18]. Such behavior occurs due to the entrainment process during the CBL growth, where clean air from Free Troposphere (FT) gets in the CBL region (negative peaks), and aerosols are transported to FT, resulting in positive peaks. Between 18:00 UTC and 19:00 UTC (Figure 11b), the skewness profile maintains the same pattern as the previous one, indicating a continuous entrainment process, which also can be observed between 19:00 UTC and 20:00 UTC (Figure 11c), where there is a higher presence of updrafts in comparison with early periods.

Figure 12 presents (from the kurtosis profiles) the change in the mixing level in the CBL region caused by the entrainment process. It varies from well-mixed (Figure 12a) to low-mixed (Figure 12b), returning to a predominance of the well-mixed again (Figure 12c). Therefore, the intense process of entrainment observed throughout the day indicates a significant entrance of air from FT in the CBL. Consequently, the decoupled layer’s plumes can contribute to increasing the concentration of PM_2.5_ in the CBL region. Significant increase tends to be observed during the night or in the following early morning due to the stable regime of the ABL and the thermal inversion above the RLH, which act as inhibitors of the dispersion process.

On this day, the AQI of PM_2.5_ was classified as moderate (>25 µg·m^−3^) at Pinheiros station, with an average daily concentration of 30 µg·m^−3^.

### 4.3. Third Day (One Day after the BB Event)

**Figure 13:**
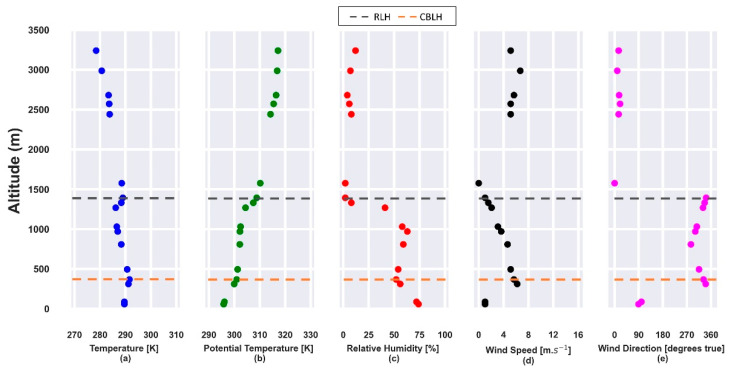
Atmospheric parameters obtained from radiosonde data on 24 July 2019, at 12:00 UTC. (**a**) Temperature [K]; (**b**) potential temperature [K]; (**c**) relative humidity [%]; (**d**) horizontal wind speed [m·s^−1^]; (**e**) wind direction [degrees true]. The gray and orange dotted lines represent the RLH and CBLH, respectively.

The CBLH and RLH, obtained from the potential temperature and RH profiles (Figure 13b,c) at 12:00 UTC, are situated around 430 m and 1450 m. Both layers are trapped by a thermal inversion (Figure 13a). Close to the surface, RH (Figure 13c) is around 77% and decreases until the RLH, where the value is lower than 45%. The horizontal wind speed profile (Figure 13d) has two maxima around 400 m and 3000 m with NNW and NE, respectively, as the predominant direction (Figure 13e).

**Figure 14:**
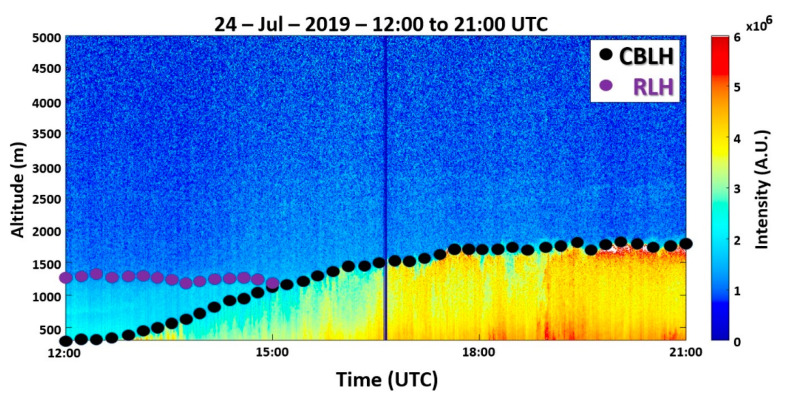
${\mathrm{RCS}}_{532}$ profiles from 12:00 UTC to 21:00 UTC on 24 July 2019. The black dots represent the CBLH, and the purple dots represent the RLH. The color bar represents the intensity of the ${\mathrm{RCS}}_{532}$.

The ${\mathrm{RCS}}_{532}$ profiles (Figure 14) indicate a well-defined ABL. The RLH oscillates between 1450 m and 1300 m, from the beginning of measurement until 15:00 UTC. The CBL has a high increase from 13:30 to 15:00 UTC (500 to 1300 m), reaching the maximum (around 1700 m) after 18:00 UTC. The decoupled aerosol layer (observed in the previous day) was not detected.

**Figure 15:**
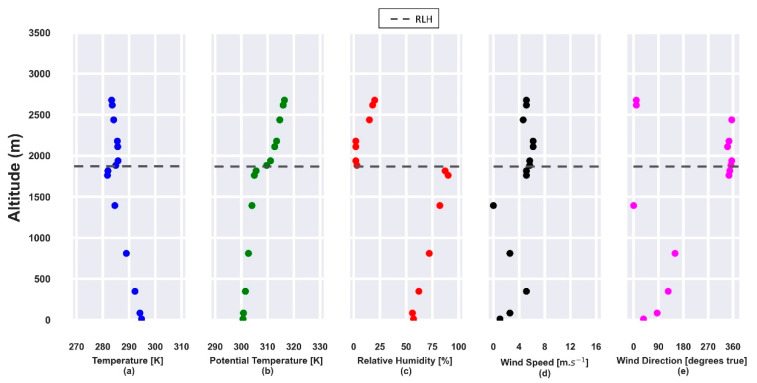
Atmospheric parameters obtained from radiosonde data on 25 July 2019, at 00:00 UTC. (**a**) Temperature [K]; (**b**) potential temperature [K]; (**c**) relative humidity [%]; (**d**) horizontal wind speed [m·s^−1^]; (**e**) wind direction [degrees true]. The gray dashed line represents the RLH.

At 00:00 UTC, from the radiosonde data, the RLH can be observed around 1900 m (Figure 15b), confined by a thermal inversion (Figure 15a). Close to the surface, the RH (Figure 15c) changes quickly from 70% to 55%, reaching around 80% at the RLH. The horizontal wind speed profile has a maximum between 2000 m and 2500 m (Figure 15d) with NNW as the predominant direction (Figure 15e).

**Figure 16:**
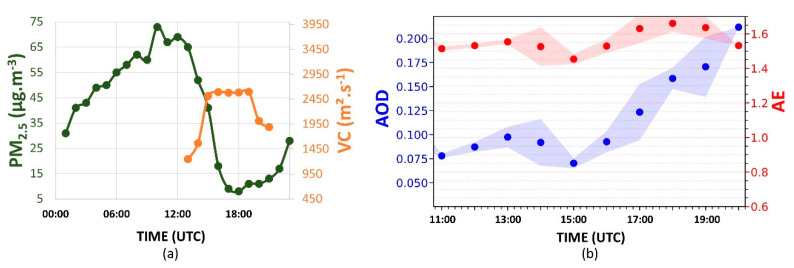
(**a**) Daily cycles of PM_2.5_ concentration (green dots) and VC (orange dots); (**b**) AOD at 500 nm (blue points) and AE (440/675) (red points) hourly averages variation from 11:00 UTC to 20:00 UTC, on 24 July 2019. The blue and red shadows represent the AOD and AE standard deviation, respectively. The AERONET data level is 1.5 for AOD and AE.

From 00:00 UTC (25 July), the PM_2.5_ concentration rises steadily (Figure 16a), reaching the maximum value (75 µg·m^−3^) at 10:00 UTC, higher than the values observed in the previous days, mainly on the day before the BB event. This difference indicates the effect that may have been caused by the entry of air from the FT because, in the regions close to the ABL, there was a decoupled aerosol layer deriving from the BB event (Figure 6). The thermal inversion and the stable regime (which inhibits the vertical mixing) limit the dispersion process; such characteristics combined with the shallow SBL could have contributed to an increase in the PM_2.5_ concentration. At 12:00 UTC, the CBLH begins to increase significantly, and the concentration of PM_2.5_ initiates to decrease, varying from 63 to 17 µg·m^−3^ between 12:00 and 16:00 UTC. Although the CBLH has a higher increase rate between 16:00 UTC and 18:00 UTC, the wind speed is low, ending in an almost constant VC value (2500 m^2^·s^−1^). From 18:00 UTC, the PM_2.5_ concentration increases again, mainly after 21:00 UTC, reaching 27 µg·m^−3^ at 23:00 UTC. As observed in the previous day, the concentration of PM_2.5_ exhibits an inverse pattern compared to the VC.

The AE (Figure 16b) presents values during the all day-cycle, close to those observed at the end of the previous day, which indicates the predominance of particles with the same small size. Also, such AE values (>1.3) were observed in other BB events indicated in the literature [37,38,39]. The AOD values (Figure 16b) are always higher than or equal to the averaged values seen in the preceding days (0.05 and 0.07 on 22 and 23 July, respectively). Moreover, such values begin to increase significantly at 15:00 UTC, reaching 0.21 at 20:00 UTC. These phenomena indicate a high aerosol accumulation in the CBL region, verified by the intensity of the ${\mathrm{RCS}}_{532}$ profile, presented in Figure 13. Consequently, the average daily value of PM_2.5_ concentration (39 µg·m^−3^) is higher than that of the previous day (30 µg·m^−3^).

On this day, the air quality index of PM_2.5_ was classified as moderate (>25 µg·m^−3^) at Pinheiros station, with an average daily concentration of 36 µg·m^−3^.

**Figure 17:**
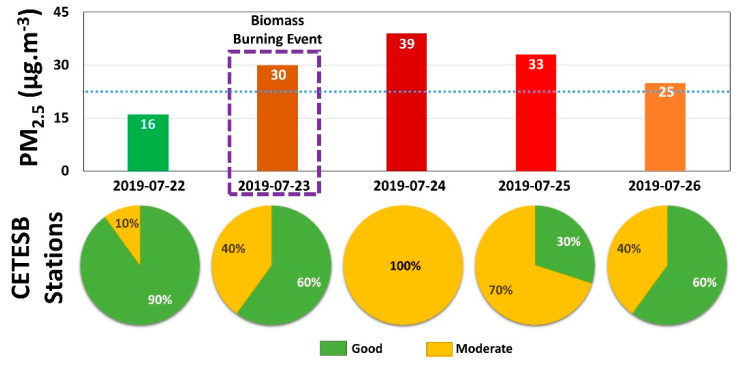
Average PM_2.5_ daily values registered at Pinheiros Environment Company of São Paulo State (CETESB) station (upper) and air quality index in CETESB stations in MASP (lower).

The upper part of Figure 17 presents the average PM_2.5_ daily value concentration variation from 22 to 26 July 2019 at *Pinheiros* CETESB station. The dotted light blue line represents the monthly average PM_2.5_ concentration value obtained in this station. On the day of the BB event, the PM_2.5_ concentration is almost double (30 µg·m^−3^) of the early day (16 µg·m^−3^). However, in the following days, the PM_2.5_ concentration increases, reaching the maximum value (39 µg·m^−3^) on 24 July (one day after the BB event) and decreasing slowly, staying above the average monthly value (24 µg·m^−3^) during four days. The lower part of Figure 17 shows the percentage of CETESB stations (in MASP) with air quality index as good (green) and moderate (yellow) between 22 to 26 July 2019. One day before the BB event, 90% of the stations indicated the AQI to PM_2.5_ as good (≤25 µg·m^−3^). On the 23 July 2019 (day of the BB event), the number of stations with AQI air quality classified as moderate (>25 µg·m^−3^) became four times larger, representing 40% of stations in MASP. One day after the BB event (24 July 2019), all CETESB stations indicated the AQI to PM_2.5_ as moderate. The air quality in the city started to improve gradually from 25 July, and on 26 July (3 days after the plume arrived), 40% of the stations still indicated the air quality as moderate.

This analysis demonstrates that the influence of BB events on the air quality is not restricted to days where the BB plume is detected. On the contrary, as CETESB sensors have their measurements limited to the surface region, this prevents layers of aerosols located in higher regions being detected. However, the ${\mathrm{RCS}}_{532}\;$skewness and kurtosis profiles allow a better understanding of the interaction process between FT and ABL so that after continuous exchanges between these two layers, it is expected that surface sensors can detect the BB event effect. Also, the thermal inversion above the RL and CBL (which can confine the aerosol) and the low values of VC (which inhibits the dispersion process) can cause an expansion, over several days, of the harmful effect from BB events in the air quality at the MASP. Similar situations, in terms of the influence of the meteorological parameters in the PM_2.5_ concentration, were also observed in other cities like Beijing [41] and Shanghai [42,43]. Therefore, only the variations of CBLH values cannot be considered the primary cause of the changes in the PM_2.5_ concentration. However, a more detailed understanding can be obtained if more atmospheric variables are analyzed (e.g., VC) together. In a comparison work, Tang et al. [20] found a low correlation between these two variables when RH is lower than 80%, which is coincident with all three days presented here, mainly in the region close to the surface. However, the VC’s variation affects the PM_2.5_ concentrations more intensively, indicating that although the entrainment process is fundamental, the horizontal wind speed also needs to be considered because it has high importance in the pollutant diffusion. Therefore, there is a negative correlation between PM_2.5_ and the VC.

## 5. Conclusions

This paper analyzes the influence of a BB event on the air quality of the MASP. On the first day of measurements (22 July 2019, one day before the BB event), an average daily PM_2.5_ concentration of 16 µg·m^−3^ was registered. The next day (23 July 2019), a decoupled aerosol layer nearby 2500 m was observed (from lidar data) which, according to lidar ratio values, (50 ± 34) sr at 532 nm and (72 ± 45) sr at 355 nm, represents a BB event. Such an argument was reinforced by the backward trajectories generated from HYSPLIT data (from regions where wildfires were detected by Aqua and GOES-16 satellites to MASP), by the wind direction obtained from radiosonde data, and by the increase of AE, which reached a similar range of values than those observed in the BB episodes registered in the literature (>1.3). Although the aerosol layer and ABL are not connected, the entrainment of air from FT in the CBL and the mixing-level variations observed from skewness and kurtosis profiles demonstrate that, gradually, the influence of the decoupled layer affects the ABL region. A significant increase in the PM_2.5_ concentration tends to be observed during the night or in the following early morning because the low values of VC and the thermal inversion above the RL inhibit the dispersion process. Consequently, in the subsequent day (24 July 2019), the PM_2.5_ concentration increased, reaching an average daily value of 39 µg·m^−3^. Unfortunately, the meteorological characteristics of the MASP winter do not favor an effective dispersion process, so that during four consecutive days, the PM_2.5_ concentration stayed above the monthly average (24 µg·m^−3^). Between 23 and 26 July 2019, 40% or more of the CETESB stations in MASP remained with air quality to PM_2.5_ as moderate so that on 24 July, all stations had the air quality classified as moderate.

The joining of remote sensing, surface monitoring stations, and backward trajectories data enabled a BB event detection. However, the air quality and concentration of PM_2.5_ are not affected because the CETESB sensors are limited to detection at the surface level. They can then indicate that an aerosol plume decoupled from the ABL does not affect the lower regions of the atmosphere. However, from the ${\mathrm{RCS}}_{532}$ skewness and kurtosis analysis, it was possible to demonstrate as they occur the exchanges between ABL and FT, so that the influence of the plume at the surface can be detected after a period of constant interaction between these two layers and, in the case of MASP, this is intensified by the thermal inversions and low VC values, to a period of three days. Therefore, the methodology applied in this work represents a new application that can be given to lidar systems and used by monitoring networks, such as the Latin American LIdar NETwork (LALINET) and European Aerosol Research LIdar NETwork (EARLINET), adding new results to the research, without the need to acquire more equipment. In the future, these analyses will be extended to other periods, and with the collaboration of other research centers, to other regions where BB events have occurred.

## Data Availability

The data presented in this study are available on request from the corresponding author.

## References

[1] IPCC (2014). Climate Change 2014: Synthesis Report. Contribution of Working Groups I, II, and III to the Fifth Assessment Report of the Intergovernmental Panel on Climate Change.

[2] Andrade M.F., Kumar P., Freitas E.D., Ynoue R.Y., Martins J., Martins L.D., Nogueira T., Perez-Martinez P., Miranda R.M., Albuquerque T. (2017). Air quality in the megacity of São Paulo: Evolution over the last 30 years and future perspectives. Atmos. Environ..

[3] Reid J.S., Lagrosas N.D., Jonsson H.H., Reid E.A., Atwood S.A., Boyd T.J., Ghate V.P., Xian P., Posselt D.J., Simpas J.B. (2016). Aerosol meteorology of Maritime Continent for the 2012 7SEAS southwest monsoon intensive study—Part 2: Philippine receptor observations of fine-scale aerosol behavior. Atmos. Chem. Phys..

[4] Lolli S., Khor W.Y., Matjafri M.Z., Lim H.S. (2019). Monsoon Season Quantitative Assessment of Biomass Burning Clear-Sky Aerosol Radiative Effect at Surface by Ground-Based Lidar Observations in Pulau Pinang, Malaysia in 2014. Remote Sens..

[5] Pani S.K., Wang S.H., Lin N.H., Lee C.T., Tsay S.C., Holben B.N., Janjai S., Hsiao T.C., Chuang M.T., Chantara S. (2016). Radiative Effect of Springtime Biomass-Burning Aerosols over Northern Indochina during 7-SEAS/BASELInE 2013 Campaign. Aerosol Air Qual. Res..

[6] Newby D.E., Mannucci P.M., Tell G.S., Baccarelli A.A., Brook R.D., Donaldson K., Forastiere F., Franchini M., Franco O.H., Graham I. (2015). Expert position paper on air pollution and cardiovascular disease. Eur. Heart J..

[7] Brook R.D., Rajagopalan S., Pope C.A.I., Brook J.R., Bhatnagar A., Diez-Roux A.V., Kaufman J. (2010). Particulate matter air pollution and cardiovascular disease, an update to the scientific statement from the American Heart Association. Circulation.

[8] Pope C.A.I., Dockery D.W. (2012). Health effects of fine particulate air pollution: Lines that connect. J. Air Waste Manag. Assoc..

[9] Landulfo E., Lopes F.J.S., Mariano G.L., Torres A.S., Jesus W.C., Nakaema W.N., Jorge M.P.P.M., Mariani R. (2010). Study of the Properties of Aerosols and the Air Quality Index Using a Backscatter Lidar System and Aeronet Sunphotometer in the City of São Paulo, Brazil. J. Air Waste Manag. Assoc..

[10] Pereira G.M., Teinilä K., Custódio D., Santos A.G., Xian H., Hillamo R., Alvez C.A., Andrade J.B., Rocha G.O., Kumar P. (2017). Particulate pollutants in the Brazilian city of São Paulo: 1-year investigation for the chemical composition and source apportionment. Atmos. Chem. Phys..

[11] Lopes F.J.S., Moreira G.A., Rodrigues P.F., Guerrero-Rascado J.L., Andrade M.F., Landulfo E. Lidar measurements of tropospheric aerosol and water vapor profiles during the winter season campaigns over the metropolitan area of Sao Paulo, Brazil. Proceedings of the SPIE 9246, Lidar Technologies, Techniques, and Measurements for Atmospheric Remote Sensing X.

[12] Noh Y.M., Müller D., Shin D.H., Lee H., Jung J.S., Lee K.H., Cribb M., Li Z., Kim Y.J. (2009). Optical and microphysical properties of severe haze and smoke aerosol measured by integrated remote sensing techniques in Gwangju, Korea. Atmos. Environ..

[13] Amiridis V., Balis D.S., Giannakaki E., Stohl A., Kazadzis S., Koukouli M.E., Zanis P. (2009). Optical characteristics of biomass burning aerosols over Southeastern Europe determined from UV-Raman lidar measurements. Atmos. Chem. Phys..

[14] Mariano G.L., Lopes F.J.S., Jorge M.P.O.M., Landulfo E. (2010). Assessment of biomass burnings activity with synergy of sunphotometric and LIDAR measurements in São Paulo, Brazil. Atmos. Res..

[15] Chan K.L. (2017). Biomass burning source and their contributions to the local air quality in Hong Kong. Sci. Total Environ..

[16] Ma X., Wang C., Han G., Ma Y., Li S., Gong W., Chen J. (2019). Regional Atmospheric Aerosol Pollution Detection Based on LiDAR Remote Sensing. Remote Sens..

[17] Moreira G.A., Guerrero-Rascado J.L., Benavent-Oltra J.A., Ortiz-Amezcua P., Román R., Bedoya-Velásquez A.E., Bravo-Aranda J.A., Olmo Reyes F.J., Landulfo E., Alados-Arboledas L. (2019). Analyzing the turbulent planetary boundary layer by remote sensing systems: The Doppler wind lidar, aerosol elastic lidar and microwave radiometer. Atmos. Chem. Phys..

[18] Moreira G.A., Lopes F.J.S., Guerrero-Rascado J.L., Silva J.J., Gomes A.A., Landulfo E., Alados-Arboledas L. (2019). Analyzing the atmospheric boundary layer using high-order moments obtained from multiwavelength lidar data: Impact of wavelength choice. Atmos. Meas. Technol..

[19] Tang G., Zhu X., Hu B., Xin J., Wang L., Münkel C., Mao G., Wang Y. (2015). Impact of emission controls on air quality in Beijing during APEC 2014: Lidar ceilometer observations. Atmos. Chem. Phys..

[20] Tang G., Zhang J., Zhu X., Song T., Münkel C., Hu B., Schäfer K., Liu Z., Zhang J., Wang L. (2016). Mixing Layer height and its implication for air pollution over Beijing, China. Atmos. Chem. Phys..

[21] Lee J., Hong J., Lee K., Hong J., Velasco E., Lim Y.J., Lee J.B., Nam K., Park J. (2019). Ceilometer Monitoring of Boundary-Layer Height and Its Application in Evaluation the Dilution Effect on Air Pollution. Bound.-Layer Meteorol..

[22] IBGE—Instituto Brasileiro de Geografia e Estatística Home Page. http://ibge.gov.br.

[23] CETESB QUALAR Home Page. http://cetesb.sp.gov.br/ar/qualar/.

[24] IAG—Estação Home Page. http://www.estacao.iag.usp.br/boletim.php.

[25] CETESB Qualidade do Ar No Estado de São Paulo 2018. https://cetesb.sp.gov.br/ar/wp-content/uploads/sites/28/2019/07/Relat%C3%B3rio-de-Qualidade-do-Ar-2018.pdf.

[26] Holben B., Eck T., Slutsker I., Tanré D., Buis J., Setzer A., Vermote E., Reagan J., Kaufman Y., Nakajima T. (1998). AERONET—A Federated Instrument Network and Data Archive for Aerosol Characterization. Remote Sens. Environ..

[27] Draxler R.R., Hess D. (1998). An overview of the HYSPLIT_4 modeling system for trajectories, dispersion, and deposition. Aust. Meteorol. Mag.

[28] INPE—Queimadas Home Page. http://queimadas.dgi.inpe.br/queimadas/portal.

[29] Stull R.B. (1988). An Introduction to Boundary Layer Meteorology.

[30] Baars H., Ansmann A., Engelmann R., Althausen D. (2008). Continuous monitoring of the boundary-layer top with lidar. Atmos. Chem. Phys..

[31] de Arruda Moreira G., Guerrero-Rascado J.L., Bravo-Aranda J.A., Foyo-Moreno I., Cazorla A., Alados I., Lyamani H., Landulfo E., Alados-Arboledas L. (2020). Study of the planetary boundary layer height in an urban environment using a combination of microwave radiometer and ceilometer. Atmos. Res..

[32] Holzworth G.C. (1964). Estimates of mean maximum mixing depths in the contiguous United States. Mon. Wheater Rev..

[33] Seidel D.J., Ao C.O., Li K. (2010). Estimating climatological planetary boundary layer heights from radiosonde observations: Comparison of methods and uncertainty analysis. J. Geophys. Res..

[34] CET Mobilidade No Sistema Viário Principal. Volumes 2018. http://www.cetsp.com.br/media/969813/relatorio-msvp-2018.pdf.

[35] Ansmann A., Riebesell M., Weitkamp C. (1990). Measurement of atmospheric aerosol extinction profiles with a Raman lidar. Opt. Lett..

[36] Ansmann A., Wandinger U., Riebesell M., Weitkamp C., Michaelis W. (1992). Independent measurement of extinction and backscatter profiles in cirrus clouds by using a combined Raman elastic-backscatter lidar. Appl. Opt..

[37] Ferrare R.A., Melfi S.H., Whiteman D.N., Evans K.D., Leifer R. (1998). Raman lidar measurements of aerosol extinction and backscattering: 1. Methods and comparisons. J. Geophys. Res..

[38] Müller D., Mattis I., Wandinger U., Ansmann A., Althausen D., Stohl A. (2005). Raman lidar observations of aged Siberian and Canadian forest fire smoke in the free troposphere over Germany in 2003: Microphysical particle characterization. J. Geophys. Res..

[39] Müller D., Ansmann A., Mattis I., Tesche M., Wandinger U., Althausen D., Pisani G. (2007). Aerosol-type-dependent lidar ratios observed with Raman lidar. J. Geophys. Res..

[40] Nicolae D., Nemuc A., Müller D., Talianu C., Vasilescu J., Belegante L., Kolgotin A. (2013). Characterization of fresh and aged biomass burning events using multiwavelength Raman lidar and mass spectrometry. J. Geophys. Res. Atmos..

[41] Xu Y., Xue W., Lei Y., Zhao Y., Cheng S., Ren Z., Huang Q. (2018). Impact of Meteorological conditions on PM_2.5_ pollution in China during winter. Atmosphere.

[42] Liang P., Zhu T., Fang Y., Li Y., Han Y., Wu Y., Hu M., Wang J. (2017). The role of meteorological conditions and pollution control strategies in reducing air pollution in Beijing during APEC 2014 and Victory parade 2015. Atmos. Chem. Phys..

[43] Xu X., Jiang Z., Li J., Chu Y., Tan W., Li C. (2020). Impacts of meteorology and emission control on the abnormally low particulate matter concentration observed during the winter of 2017. Atmos. Environ..

